# Computational Tools for *Brassica–Arabidopsis* Comparative Genomics

**DOI:** 10.1002/cfg.463

**Published:** 2005-04

**Authors:** Paul Beckett, Ian Bancroft, Martin Trick

**Affiliations:** 1 Computational Biology Group John Innes Centre Norwich Research Park Norwich NR4 7UH UK; 2 Crop Genetics Department John Innes Centre Norwich Research Park Norwich NR4 7UH UK

## Abstract

Recent advances, such as the availability of extensive genome survey sequence (GSS) data and draft physical maps, are radically transforming the means by which we can dissect *Brassica* genome structure and systematically relate it to the *Arabidopsis* model. Hitherto, our view of the co-linearities between these closely related genomes had been largely inferred from comparative RFLP data, necessitating substantial interpolation and expert interpretation. Sequencing of the *Brassica rapa* genome by the Multinational *Brassica* Genome Project will, however, enable an entirely computational approach to this problem. Meanwhile we have been developing databases and bioinformatics tools to support our work in *Brassica* comparative genomics, including a recently completed draft physical map of *B. rapa* integrated with anchor probes derived from the *Arabidopsis* genome sequence. We are also exploring new ways to display the emerging *Brassica–Arabidopsis* sequence homology data. We have mapped all publicly available Brassica sequences *in silico* to the *Arabidopsis* TIGR v5 genome sequence and published this in the ATIDB database that uses Generic Genome Browser (GBrowse). This *in silico* approach potentially identifies all paralogous sequences and so we colour-code the significance of the mappings and offer an integrated, real-time multiple alignment tool to partition them into paralogous groups. The MySQL database driving GBrowse can also be directly interrogated, using the powerful API offered by the Perl Bio∷DB∷GFF methods, facilitating a wide range of data-mining possibilities.


**Comparative and Functional Genomics**
*Comp Funct Genom* 2005; **6**: 147–152.
Published online in Wiley InterScience (www.interscience.wiley.com). **DOI:** 10.1002/cfg.463

**Conference Paper**

### Computational tools for *Brassica–Arabidopsis* comparative genomics

Paul Beckett[1], Ian Bancroft[2] and Martin Trick[1]*
[1]*Computational Biology Group, John Innes Centre, Norwich Research Park, Norwich NR4 7UH, UK*
[2]*Crop Genetics Department, John Innes Centre, Norwich Research Park, Norwich NR4 7UH, UK*

**Correspondence to: Martin Trick, Computational Biology Group, John Innes Centre, Norwich Research Park, Norwich NR4 7UH, UK. E-mail: martin.trick@bbsrc.ac.uk*

### Abstract

**Recent advances, such as the availability of extensive genome survey sequence (GSS) data and draft physical maps, are radically transforming the means by which we can dissect *Brassica* genome structure and systematically relate it to the *Arabidopsis* model. Hitherto, our view of the co-linearities between these closely related genomes had been largely inferred from comparative RFLP data, necessitating substantial interpolation and expert interpretation. Sequencing of the *Brassica rapa* genome by the Multinational *Brassica* Genome Project will, however, enable an entirely computational approach to this problem. Meanwhile we have been developing databases and bioinformatics tools to support our work in *Brassica* comparative genomics, including a recently completed draft physical map of *B. rapa* integrated with anchor probes derived from the *Arabidopsis* genome sequence. We are also exploring new ways to display the emerging *Brassica–Arabidopsis* sequence homology data. We have mapped all publicly available *Brassica* sequences *in silico* to the *Arabidopsis* TIGR v5 genome sequence and published this in the ATIDB database that uses Generic Genome Browser (GBrowse). This *in silico* approach potentially identifies all paralogous sequences and so we colour-code the significance of the mappings and offer an integrated, real-time multiple alignment tool to partition them into paralogous groups. The MySQL database driving GBrowse can also be directly interrogated, using the powerful API offered by the Perl Bio::DB::GFF methods, facilitating a wide range of data-mining possibilities.** **Copyright © 2005 John Wiley & Sons, Ltd.**

Received: 13 January 2005
Accepted: 1 February 2005

**Keywords:** ***Brassica*; *Arabidopsis*; bacterial artificial chromosome (BAC); physical mapping; ATIDB; GBrowse**

### Introduction

The *Brassica* species complex occupies a pivotal and potentially exploitable position with respect to that of the model crucifer plant *Arabidopsis thaliana*, whose complete genome has been sequenced [12]. The genus *Brassica*, from which key oilseed, vegetable and fodder crops have been domesticated, is evolutionarily closely related to *Arabidopsis*, both being members of the family *Brassicaceae*, which diverged 14–20 million years ago [14]. This is manifested by the average 89% sequence identity and conserved structures shared by homologous gene-coding regions [2,7]. Although the genomes show extensive synteny, they nevertheless bear the hallmarks of complex evolutionary histories of polyploidization and karyotypic rearrangement. Strategies for gene isolation that exploit the relationship depend on the extent to which microsynteny is generally conserved. As the nature of the model-crop dataset evolves, with the coming sequencing of the

Copyright © 2005 John Wiley & Sons, Ltd.

*Brassica rapa* genome, so will the computational tools we use to mine it.

### Computational inferences of genome structures

Our earliest attempts to provide for computational discovery of structural genomic patterns were based on RFLP data, e.g. the duplications within the amphidiploid *Brassica napus* genome — both homologies between the constituent A and C genomes and paralogies within each — could be revealed through the GridMap graphical display engine [8], driven by a Perl adaptor which, in turn, queried the BrassicaDB database [1] in real time (Figure 1a). This database contains genetic map objects corresponding to the linkage groups, with the names of loci and their observed recombination distances in centiMorgans (cM). Since we can systematize the naming of multiple loci detected by the same RFLP probe, implying homology through sequence conservation, it is possible to write scripts to automatically discover this relationship through regular expression operators on character strings; e.g. 'pW100a' would be a paralogue of 'pW100b'. Similar methods were used to illustrate the relationship between the *B. napus* genetic map and physical maps of *Arabidopsis* chromosomes (Figure 1b), this time with the PairwiseComparativeMap applet [5] supplying the graphical layer. Clearly these techniques were severely limited, both by the burden of the human-supervised curation needed to populate the datasets and the resolution offered by the underlying data itself. The prospect of an alignment between a finished *Brassica* genome sequence and that of *Arabidopsis* offers a new dimension in unsupervised pattern discovery at the sub-kilobase level.

### A draft *Brassica rapa* physical map

Computational approaches were adopted to facilitate the construction of draft physical maps for the diploid *Brassica* species, *B. rapa* and *B. oleracea*,

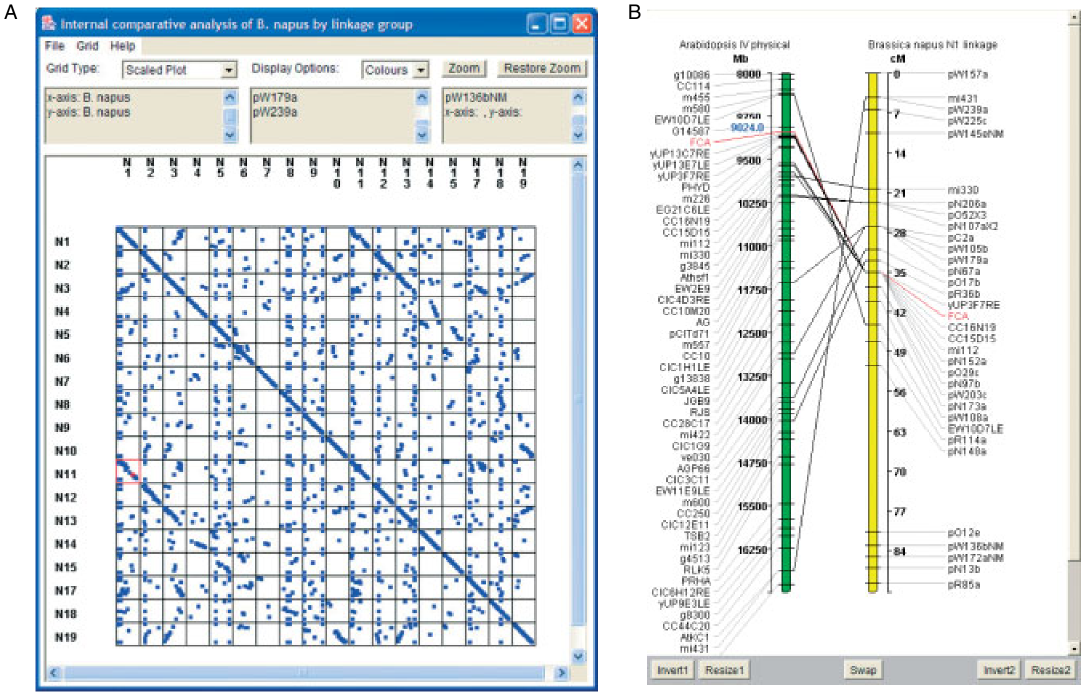

**Figure 1.** (A) GridMap display illustrating the internal genome structure of *B. napus* based on name matching of mapped genetic loci. The highlighted box, corresponding to the N1–N11 linkage group comparison, shows almost complete homology of these chromosomes. An inverted paralogy connecting N1 with N3 is also discernible. (B) PairwiseComparativeMap display illustrating inverted synteny between *B. napus* linkage group N1 and a segment of *Arabidopsis* chromosome 4

Copyright © 2005 John Wiley & Sons, Ltd. 

that were to be integrated with the *Arabidopsis* genome sequence. Until the finished *B. rapa* genome sequence becomes available, these data (**http://brassica.bbsrc.ac.uk/IGF/**) provide a useful, yet inevitably incomplete, comparative route between model and crop. This collaborative UK project, between groups at the John Innes Centre, HRI Wellesbourne and the Universities of Bath and Birmingham, involved mapping of BAC libraries for the two diploid genomes by fingerprinted contig analysis using Image [13] and FPC [10], followed by colony hybridization screening with about 1300 *Arabidopsis* gene sequence probes. For this we devised a software pipeline to identify candidate gene sequences from the *Arabidopsis* genome at approximately 100 kb intervals. These needed to be derived from exons to maximize the heterologous hybridization signals and to define PCR amplicons of 250–500 bp. Most importantly, we needed to ensure these amplicons were essentially unique within the *Arabidopsis* genome in order to reduce the complexity of the results obtained from the duplicated *Brassica* genome, thus increasing the specificity of the subsequent anchoring of the contigs. This latter was tested with the WU-BLASTN program [6], querying candidate sequences against the *Arabidopsis* genome and selecting for unequivocally unique hits at an $E$-value cut-off of $10^{-10}$. If passed, the candidate sequence was then passed to the Primer3 program [9] to select primers for PCR amplification.

The results from this project are available through a web interface to a MySQL database at **http://brassica.bbsrc.ac.uk/IGF/**. The user can query the database to discover contigs on a number of criteria, such as origin and position of anchor probes, their annotated function, etc. The user can elect to receive the results in a number of formats; simple static tables written to their browser window, dynamic tables that allow efficient, client-side sorting or as a fully functional Excel worksheet. The names of the database objects returned are clickable hyperlinks that retrieve further details on the object, usually in the form of text but, in the case of BAC contigs, interactive graphical displays (Figure 2).

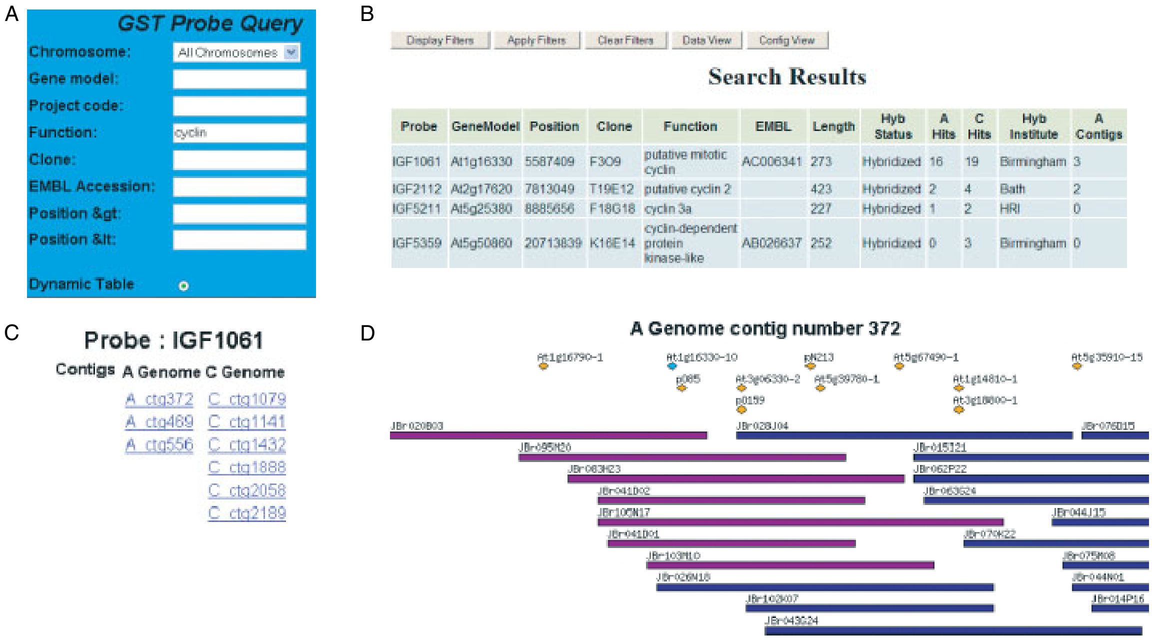

**Figure 2.** An illustration of the query capabilities of the project database. (A) an initial query ('cyclin'); (B) search results for anchor probes with the term 'cyclin' in their annotation; (C) selection of one such probe, At1g16330 (IGF1061), listing all available contigs; (D) the display of *B. rapa* contig A_ctg372. Clicking on the probe icon here colourizes all the constituent clones that co-hybridize. In this case, therefore, the user can be highly confident that this contig contains a *Brassica* homologue to At1g16330

### Mapping *Brassica* homologues to the *Arabidopsis* genome

Until the international *Brassica* sequencing initiative is completed, and probably thereafter, there should be considerable utility in performing *in silico* mappings of *Brassica* sequences to the *Arabidopsis* pseudochromosomes and making the results publicly available. Almost all functional genomics assignments are likely to be made in the model and so, at present, this seems to be the natural and most productive way in which to present the data. There does remain a requirement for a reverse route to facilitate identification of candidate genes underlying QTL phenotypes in the crop, something which genetic mapping initiatives that offer integration with the *Arabidopsis* genome sequence can address.

We used WU-BLASTN, implemented on a 20 dual-CPU node Linux cluster, to query a total of 596 930 *Brassica* GSS and 50 858 EST sequences against the *Arabidopsis* TIGR v5 pseudochromosome sequences. A significance threshold of $E \leqslant 1 \times 10^{-10}$ was applied, translating to a sequence identity of around 60% over the typical HSPs recovered in this exercise. An hspsepSmax parameter of 1000 was invoked in order to restrict the BLAST algorithm's HSP extension behaviour when used against very large database sequences.

This *in silico* method appeared to work well, with the high level of sequence conservation between the genomes allowing facile identification of homologous EST sequences (hit rate of 89%) and also a significant proportion of the GSS sequences (51%). Generally, the hit densities for each *Arabidopsis* chromosome correlated well with the uniform annotated gene density of about 250 gene models/Mb. However, we did observe an apparent skew for *Arabidopsis* chromosome 2, which seemed to be under-represented in *Brassica* EST hits and over-represented with GSS hits. We are investigating possible causes of this effect; one might be a preponderance of conserved transposon or other repeated sequences.

There are *caveats* with regard to this approach. The principal caution is that, while the genome coverage provided by the *B. oleracea* GSS accessions remains incomplete, our BLAST analysis will sometimes associate a somewhat diverged paralogue with a given *Arabidopsis* segment, where a more conserved copy is yet to be discovered in *Brassica*. Users browsing our digested data are alerted to this, as described below. Conversely, and perhaps more seriously, our parsing algorithm is relatively simplistic and currently does not capture any highly significant, secondary hits that might reflect the *Arabidopsis* genome's evolutionary history of segmental duplication [12].

We are applying the same methodology to the new wave of *Brassica* GSS accessions supplied by paired BAC-end sequence reads of the *B. rapa* KBrH library that is to form the template for the international sequencing programme. Hypothesizing that a fraction of these paired reads should support an underlying microsynteny with *Arabidopsis* over the 100–300 kbp range, we post-processed the BLAST data to mark up those consistent pairs as belonging to candidate co-linear clones and the remaining orphans as singleton GSS features. For instance, we found that 56% of BAC-end sequences, drawn from a sample of 2960 KBrH clones, gave significant BLAST hits, similar to the results described above for the *B. oleracea* GSSs. In this pilot experiment, 20% of the clones were 'mapped' successfully according to our criteria, but 18% had ends that mapped to different chromosomes and 6% had anomalous inferred insert sizes (assuming 0.5–3.0-fold relative intergenic expansion). The remaining clones did not have good quality sequence reads for each end and were excluded from this analysis. It remains to be seen whether the 24% that fall outside our first approximation criteria are real or artefacts. Some may reflect anomalies arising from duplications but, intuitively, many should be real, as the karyotypes imply chromosome fusion/fission events and RFLP-based comparative analysis [7] has certainly indicated substantial accumulation of such breakpoints, especially around the centromeres. The question is how fine-grained these disruptions to synteny will turn out to be, something which only the finished *B. rapa* sequence can answer.

### GBrowse viewer and data-mining with Bio::DB::GFF

This basic *in silico* mapping approach has been used by a number of groups, but different database applications and browser interfaces have been adopted for displaying the results. For instance,

Copyright © 2005 John Wiley & Sons, Ltd. 

the AAFC Saskatoon Research Centre has developed its own *Brassica–Arabidopsis* Comparative Genome Viewer (**http://brassica.agr.gc.ca/navigation/viewer_e.shtml**) to display its *B. napus* ESTs mapped to TIGR v5. We chose to extend a pre-existing resource, already dedicated to the biology of the model organism. The same philosophy has been adopted by the Plant Biotechnology Centre in Victoria, Australia, in collaboration with the UK Nottingham *Arabidopsis* Stock Centre, in adding *Brassica* data to the AtEnsembl viewer (**http://atensembl.arabidopsis.info**).

We have added the *Brassica* data described above to the ATIDB database resource (**http://atidb.org**) that is developed and maintained at the John Innes Centre. ATIDB is a repository for *Arabidopsis* functional genomics data, such as gene knock-outs and gene traps generated by insertion mutagenesis programmes, and is fully integrated with the TIGR v5 genome sequence, associated gene model annotation and the Gene Ontology schema. ATIDB uses the GMOD group's GBrowse genome viewer [11] as the application to display the genome features. GBrowse is an open-source, highly configurable and extensible software system. A unique feature is the ability for users to transiently upload their own data and thus overlay an in-house, proprietary view of the genome

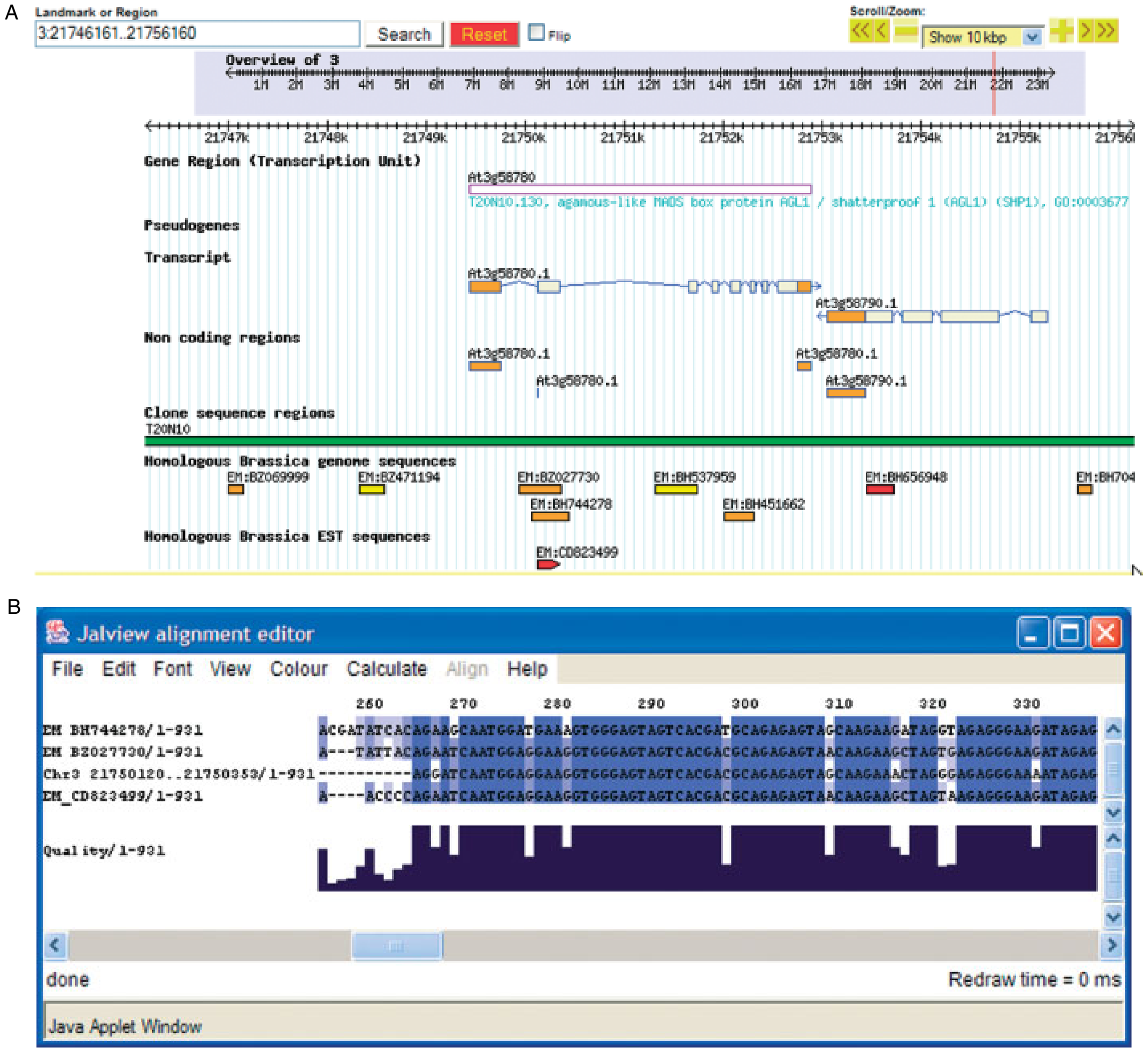

**Figure 3.** (A) An example screenshot of the ATIDB database illustrating *Brassica* homology features mapped *in silico* and (B) their real time multiple alignment with the *Arabidopsis* sequence

Copyright © 2005 John Wiley & Sons, Ltd. 

onto the public dataset. The ATIDB implementation of GBrowse employs an adaptor to a MySQL database containing the genome sequence and feature data using the Bio::DB::GFF schemata that are highly optimized for querying on feature names or genome locations. All that was required to load the *Brassica* data into ATIDB was to prepare from the BLAST analysis a tab-delimited file in GFF format recording the *in silico*-derived coordinates of the feature homologies and to apply a simple reconfiguration. We included in the GFF file sequence identity scores parsed from the BLAST HSPs. This information is used by GBrowse's graphical layer to colour-code each alignment feature. In many cases this makes the identification of differentially conserved *Brassica* paralogues readily apparent. In addition we have added methods by which clicking on any *Brassica* feature launches a real-time multiple alignment for all sequences overlapping the selected region. This is powered by ClustalW [3] on our server and is graphically rendered by deploying the Jalview applet [4] to the client (Figure 3). This system allows visualization of the sequence relationships within paralogous sets and also leverages the power of comparative genomics to inform on the accuracy of the current gene model annotation.

Finally, as well as the web interface suitable for casual browsing and searching, the underlying MySQL database is also accessible to programmers through the rich and versatile Perl Bio::DB::GFF interface that allows sophisticated data-mining. For instance, by invoking these methods, it takes only about 20 lines of Perl code to systematically trawl the mapped *Brassica* features for those that lie outside currently annotated regions of the *Arabidopsis* genome. This facilitates a useful approach to gene discovery and re-annotation, powered by comparative genomics.

### Future perspectives

We are entering an exciting time for comparative genomics in a key model-crop system and the availability of a finished *Brassica* genome sequence will both shape and test the informatics tools we use to analyse it. It is likely, however, that systems which facilitate reverse navigation, from genetic marker to sequence, will assume a greater significance as scientists tackle the important agronomic issues with QTL analyses.

### Acknowledgements

This work was supported by BBSRC Grant No. 208/IGF 12448 and the BBSRC's strategic grant to the John Innes Centre. We thank Sean Walsh for collaboration and for hosting the *Brassica* data in ATIDB. This paper is dedicated to the fond memory of Dr George Murphy.

### References

1. Anderson ML, Cardle L, Cartinhour S, *et al*. 2000. UK CropNet: A collection of databases and bioinformatics resources for crop plant genomics. *Nucleic Acids Res* **28**: 104–107.
2. Cavell AC, Lydiate DJ, Parkin IAP, Dean C, Trick M. 1998. A 30 centiMorgan segment of *Arabidopsis thaliana* chromosome 4 has six co-linear homologues within the *Brassica napus* genome. *Genome* **41**: 62–69.
3. Chenna R, Sugawara H, Koike T, *et al*. 2003. Multiple sequence alignment with the Clustal series of programs. *Nucleic Acids Res* **31**: 3497–3500.
4. Clamp M, Cuff J, Searle SM, Barton GJ. 2004. The Jalview Java Alignment Editor. *Bioinformatics* **12**: 426–427.
5. Dickson J, McWilliam H. 2000. PCM: **http://jic-bioinfo.bbsrc.ac.uk/JavaDocs/uk/ac/CropNet/PairwiseUser/package-summary.html**.
6. Gish W. 1996–2004. WU-BLAST: **http://blast.wustl.edu**.
7. Parkin IAP, Lydiate DJ, Trick M. 2002. Assessing the level of co-linearity between *Arabidopsis thaliana* and *Brassica napus* for *A. thaliana* chromosome 5. *Genome* **45**: 356–366.
8. Priestly M, Dickson J, Dicks J. 2002. GridMap: **http://jic-bioinfo.bbsrc.ac.uk/bioinformatics-research/software/Grid_Map/**.
9. Rozen S, Skaletsky HJ. 2000. Primer3 on the WWW for general users and for biologist programmers. In: *Bioinformatics Methods and Protocols: Methods in Molecular Biology*, Krawetz S, Misener S (eds). Humana: Totowa, NJ; 365–386.
10. Soderlund C, Humphrey S, Dunhum A, French L. 2000. Contigs built with fingerprints, markers and FPC V4.7. *Genome Res* **10**: 1772–1787.
11. Stein LD, Mungall C, Shu S, *et al*. 2002. The generic genome browser: a building block for a model organism system database. *Genome Res* **12**: 1599–1610.
12. The *Arabidopsis* Genome Initiative. 2000. Analysis of the genome sequence of the flowering plant *Arabidopsis thaliana*. *Nature* **408**: 796–815.
13. Wobus F, *et al*. 2001. Image: **http://www.sanger.ac.uk/Software/Image/**.
14. Yang YW, Lai KN, Tai PY, Li WH. 1999. Rates of nucleotide substitution in angiosperm mitochondrial DNA sequences and dates of divergence between *Brassica* and other angiosperm lineages. *J Mol Evol* **48**: 597–604.

Copyright © 2005 John Wiley & Sons, Ltd.
